# Sex and hypertensive organ damage: stroke

**DOI:** 10.1038/s41371-023-00830-0

**Published:** 2023-04-14

**Authors:** Jesse Dawson, Alexander MacDonald

**Affiliations:** School of Cardiovascular and Metabolic Health, College of Medical, Veterinary & Life Sciences, University of Glasgow, Queen Elizabeth University Hospital, Glasgow, UK

**Keywords:** Cardiovascular diseases, Health care

## Abstract

Stroke is a common cause of death and disability in both men and women. Differences in the incidence, presenting features and outcome after stroke have been reported between men and women. The global lifetime risk of stroke of approximately 25% is similar in men and women, although in women, the first cardiovascular event is more likely to be stroke than in men. Concerningly, there are reports of underuse of some treatments in women, although these differences may be diminishing over time. In addition, there are specific clinical challenges that can arise in women with stroke, such as stroke in people taking hormonal therapy, and stroke during pregnancy and stroke in the post-partum period. This review will cover these areas highlighting important differences and areas for future research. We found there are important differences in incidence of stroke, which differ by age. Further, there is concerning evidence that some treatments such as intravenous thrombolysis are underused in women. While there may be some differences in the relative effectiveness of treatments such as antiplatelet therapy and blood pressure reduction between men and women, for most aspects of stroke care, benefit is clear in both men and women and the emphasis must be on more equitable access. There is limited evidence to inform decision making during pregnancy and the post-partum period, but guidelines now exist and further research is needed in these areas.

## Introduction

Stroke is a common cause of death and disability in both men and women. It is increasingly recognised that incidence and outcome of stroke differ in men and women, as may the response to commonly used treatments. In addition, there are clinical challenges which are specific to women, such as stroke in people taking contraceptive and hormone replacement therapy and stroke during pregnancy or in the post-partum period. This review will cover these areas highlighting important differences and areas for future research. The review will focus on sex related differences rather than specific sex-independent gender related differences. This is largely because of a paucity of data on gender specific differences in stroke and there is a need for further research in this area. A summary is shown in Table 1.

**Table 1:** Summary of Sex Related Differences in Stroke.

Stroke Characteristics	Sex Related Differences	Recommendations/Areas for Future Research/Areas Lacking Evidence
Incidence and presentation	Age-specific incidence is higher in men, but more strokes occur in women. Women are less likely to present within four hours to stroke services, and more likely to present with less typical symptoms.	Data on gender specific differences in stroke is scarce with a need for further research. There is an apparent opportunity to improve rapid identification of stroke in women.
Outcomes and treatment access	Women have greater rates of morbidity and worse quality of life after stroke. Anticoagulation, thrombolytic therapy, and carotid endarterectomy are less commonly used in women, although the gap is narrowing over time.	Sex related disparities in mortality rates have been inconsistent between studies. More equitable access to treatment must be a priority, particularly where comparable efficacy has been demonstrated between the sexes.
Prevention	Primary prevention with aspirin reduces the risk of stroke in women but not men, although this was accompanied by a significant increased risk of gastrointestinal bleeding in women. Carotid endarterectomy appears more beneficial in men than women. However, under-representation of women was an issue in these studies.	Women have been consistently under-represented in preventative trials. In future, emphasis must be placed on ensuring greater representation of women in clinical trials to definitively establish any sex related differences. Current guidelines do not specify blood pressure targets by sex; further research is needed in this area.
Acute treatments	Women have been shown to be more likely to exhibit poor functional outcomes following intravenous thrombolysis than men.	The current conflicting evidence in sex differences in outcomes following endovascular thrombectomy warrants further research.
Clinical challenges specific to women	Hormone replacement therapy does not reduce the risk of stroke. Combined oral contraceptive treatment use increases the risk of stroke by more than two-fold. In pregnancy and the post-partum period, guidelines recommend that thrombolysis or thrombectomy (preferred) can be used in eligible patients after assessing the benefit/risk profile on an individual basis.	Data regarding progesterone-only contraceptives is limited, but they are the more appropriate choice of oral contraception in high-risk individuals. Evidence to inform decision making during pregnancy and the post-partum period is limited with research needed in this area.

## Incidence of stroke in men and women and presenting features

Age specific incidence of stroke is higher in men but more strokes, and more than half of stroke related deaths, occur in women [1]. The global lifetime risk of stroke is broadly similar between men and women (approximately 25%) although there are regional differences, with the biggest difference in rates being seen in China (41% in men vs. 37% in women) and with higher lifetime risk of stroke in women in North America, parts of Europe and in Africa [2]. Data from the Rotterdam study show that in a European population, the first cardiovascular event in women is likely to be stroke, whereas in men, it is likely to be a consequence of coronary heart disease [3]. Sex related differences in type of stroke have been reported with women having a higher incidence and case fatality of subarachnoid haemorrhage [4]. While overall, some studies report a similar risk of stroke or TIA in men and women, this appears to differ by age. Women have been reported to have a higher incidence of stroke at a younger age (<30 years) but a lower incidence in the middle and early elderly years (40–79 years) [5].

There are several plausible explanations for why the incidence of stroke may differ in men and women. First, women have a longer life expectancy and hence accumulate more stroke events. In addition, the relationship between many common cardiovascular risk factors and stroke differs in men and women. A recent analysis of data from the UK Biobank showed that the relationship between blood pressure, body mass, lipids, diabetes, and atrial fibrillation and any stroke was similar in men and women but hypertension, smoking, and poor socioeconomic status were more strongly associated with risk in women [6]. Although body mass was similarly related with risk of any stroke, it was more strongly related with risk of ischaemic stroke in women. It is unclear if some of these differences are explained by differences in access to treatment or reflect true biological differences.

Several studies have reported that women are more likely to present with less typical symptoms of stroke such as headache and cognitive disturbance. However, in a recent study, there were no observed differences in presenting symptoms between men and women, yet women were more likely to receive a diagnosis of stroke mimic [7]. This suggests there is an opportunity to improve rapid identification of stroke in women.

## Outcome and access to treatment following stroke in men and women

Several studies have explored the relationship between sex and outcome after stroke, often with inconsistent findings. An individual patient data meta-analysis of 13 studies found that women had a higher mortality than men at one and five years after stroke [8]. However, this difference did not persist following adjustment of confounding factors including age, where women had a lower mortality [8]. A recently published study included data on 19,652 people enrolled in 5 randomised studies. Women had higher survival at 3–6 months but a higher rate of disability and worse quality of life [9]. Better survival in women has also been reported in other studies during longer term (3 years) follow up [10]. Whether the reported higher disability and worse outcomes simply reflects an effect of survivor bias or a higher pre-morbid level of disability in these trials is unclear. However, differences in access to treatments were also described including a lower likelihood of intubation and admission to intensive care. It has previously been reported that women have a lower likelihood of receiving thrombolytic therapy (odds ratio (OR) 0.8, 95% confidence interval (CI) 0.7 to 0.9) and of presenting within four hours to stroke services (OR 0.8, 95% CI 0.7 to 0.9) [11]. Once the delay in presentation was accounted for, the rates of thrombolytic therapy were similar in men and women in this study. Possible explanations for delayed presentation include an older age and higher likelihood of living alone [11]. A systematic review and meta-analysis of 24 eligible trials published between 2008 and 2018 again found lower odds of receiving thrombolytic therapy in women (OR 0.87, 95% CI 0.82 to 0.93) but suggested that the size of the difference in thrombolysis rates had diminished over time [12], a fact supported by older studies where the difference was higher [13]. Other studies have reported that the overall quality of stroke care is less in women [14].

## Effect of treatments for stroke and prevention of stroke in men and women

### Primary prevention with aspirin

The use of aspirin for primary prevention of cardiovascular disease is no longer advised in many guidelines. However, the Women’s Health Study suggested a beneficial effect of aspirin in risk of stroke in women [15]. This topic has recently been addressed in a systematic review and meta-analysis of 11 randomised controlled clinical trials (135,641 participants) [16]. Differences in the effect of aspirin on primary stroke prevention were evident between men and women. A significant reduction in the risk of stroke was observed with aspirin therapy in women (OR 0.85, 95% CI 0.73 to 0.99, *p* = 0.03), driven by a decreased risk of ischaemic stroke (OR 0.76, 95% CI 0.63 to 0.93, *p* = 0.008). There was a nonsignificant increased risk of haemorrhagic stroke (OR 1.78, 95% CI 0.61 to 5.19, *p* = 0.29) with aspirin in women. In contrast, aspirin therapy did not significantly alter ischaemic stroke risk in males (OR 0.94, 95% CI 0.67 to 1.32, *p* = 0.72) but increased risk of haemorrhagic stroke (OR 1.99, 95% CI 0.99 to 4.03, *p* = 0.05). Aspirin therapy increased risk of major bleeding in both men and women, although a significant increase in risk of gastrointestinal bleeding with aspirin was only seen in women. There were also sex-based differences in the risk of myocardial infarction (MI) with aspirin treatment. There was no reduction in the risk of MI in women (OR 0.92, 95% CI 0.77 to 1.11, *p* = 0.38) but there was a significant reduction in men (OR 0.68, 95% CI 0.58 to 0.81, *p* < 0.0001) [16].

These differential effects of aspirin in men and women may be attributable to underlying differences in platelet function influenced by the actions of sex hormones. For example, platelet function is known to be influenced by testosterone and changes of the ovarian cycle [17, 18]. Overall, the net value of aspirin as a primary preventative agent remains debatable, with the protective benefit of aspirin in women at least partially offset by the risk of major bleeding. Furthermore, it is important to note that many trials of aspirin for primary prevention of cardiovascular disease were conducted before primary prevention with blood pressure reduction and lipid lowering were commonplace.

### Atrial fibrillation and anticoagulation

Atrial fibrillation (AF) is a major modifiable risk factor for stroke. It has been reported that anticoagulation for thromboembolic prophylaxis in AF is less commonly used in eligible women than in eligible men (88.0% vs. 89.7% respectively; adjusted OR 0.93, 95% CI 0.88 to 0.98, *p* = 0.008) [19]. This is despite clear evidence that anticoagulation is as effective, or perhaps even more effective, in women. In one study, warfarin reduced stroke risk in men by 60% (35–76%) and in women by 84% (55–95%) [20]. A systematic review and meta-analysis failed to identify any sex differences in the risk of stroke or systemic embolism in people with AF receiving direct oral anticoagulants. However, major bleeding rates were found to be significantly less frequent in women (OR 0.84, 95% CI 0.75 to 0.96, *p* = 0.007) [21].

### Thrombolytic therapy for acute ischaemic stroke

As described above, women are reported to be less likely to receive thrombolytic therapy for ischaemic stroke than men. There are several possible explanations. Compared with men, women with stroke are more likely to be older, widowed and living alone [22]. Consequently, women may have a lower likelihood of being admitted within the timeframe required for thrombolysis. In addition, women may be more likely to present with atypical stroke symptoms, which could also cause delay [12, 13]. Time delays are probably the critical factor. In meta-analyses looking specifically at the subgroups of patients eligible for thrombolysis (defined by arrival time and contraindications), although lower odds of treatment in women were still observed, the estimates were no longer statistically significant [12, 13].

Uncertainty remains with regards to sex-specific differences in outcomes following thrombolysis. A meta-analysis of 16 studies (60,159 patients) found that women were more likely to exhibit poor functional outcomes (modified Rankin Scale 3–6) compared to men after intravenous thrombolysis (risk ratio (RR) 1.24, 95% CI 1.11 to 1.36, *p* < 0.001) [23]. Higher levels of plasminogen activator inhibitor-1 in women, an independent predictor of thrombolysis resistance [24], is one possible explanation. In contrast, there were no sex differences apparent in outcomes after intra-arterial thrombolysis. No differences were noted between men and women in rates of symptomatic intracranial haemorrhage (ICH) [23]. However, interstudy heterogeneity was apparent and the potential for confounding bias must be considered.

### Endovascular thrombectomy for acute ischaemic stroke

Rates of thrombectomy appear comparable in men and women [22], although it remains unclear whether outcomes differ. A recent systematic review and meta-analysis of 33 studies (7335 participants) found that men undergoing endovascular thrombectomy for large-vessel occlusions had higher odds of better 90-day functional outcome (modified Rankin Scale 0–2) than women (OR 1.29, 95% CI 1.09 to 1.53, *p* < 0.001) [25]. No differences were seen in recanalization rates, 90-day mortality, and symptomatic ICH. However, a more recent study found opposing results: women treated with mechanical thrombectomy had greater probability of achieving complete recanalization and functional independence at 3 months, combined with a lower risk of death compared to men [26].

### Secondary prevention with antiplatelet therapy

In contrast to primary stroke prevention, aspirin seems to confer similar benefit for secondary stroke prevention in men and women. The Antithrombotic Trialists’ Collaboration conducted a meta-analysis of 16 secondary prevention trials (17,000 participants) that compared long-term aspirin to control therapy. Aspirin led to a 19% reduction in risk of stroke (RR 0.81, 95% CI 0.71 to 0.92), with similar magnitude of effect in men and women [27].

Another meta-analysis investigated sex-specific differences in the efficacy of clopidogrel for the prevention of cardiovascular events [28]. Five randomised trials involving 79,613 patients (30% women) were included. While clopidogrel was associated with a significant decrease in the odds of stroke in men (OR 0.83, 95% CI 0.71 to 0.96), the 9% risk reduction observed in women was not statistically significant (OR 0.91, 95% CI 0.69 to 1.21). However, there was no evidence of statistical heterogeneity in the pooled analyses between males and females for stroke (*p* = 0.552), implying that the difference could be explained by chance or a lack of study power. No significant treatment interactions between men and women were observed in the trials of dual antiplatelet therapy in people with minor stroke and TIA, suggesting this is equally effective in men and women [29].

### Lipid lowering

It is well established that lipid lowering therapy is an effective intervention in the secondary prevention of major cardiovascular events in both men and women. However, whether effectiveness differs in men and women is debated. The Cholesterol Treatment Trialists’ Collaboration meta-analysis of 174,000 participants from 27 trials found comparable efficacy of statin therapy for the prevention of stroke in men and women [30]. This study included both primary and secondary prevention trials. A prior meta-analysis, consisting of only secondary prevention trials (11 trials with 43,193 participants), found no significant benefit on stroke risk in women (RR 0.92, 95% CI 0.76 to 1.10) but a significant effect was seen in men (RR 0.81, 95% CI 0.72 to 0.92) [31]. This difference may have been due to fewer data in women, who made up only 20% of the population analysed.

### Antihypertensive treatment

Recently, there has been debate concerning whether blood pressure targets should be sex specific. This is in part because it is reported that the benefit of intensive blood pressure reduction in women appear less than in men [32] and some observational studies suggest a lower blood pressure level is needed in women for maximum benefit. Currently guidelines do not contain sex specific recommendations, but further research is needed in this area. In addition, it has recently been reported that brachial blood pressure may underestimate central blood pressure in women [33], which further supports the need to assess lower blood pressure targets.

A meta-analysis of 31 randomised blood pressure lowering clinical trials (with 103,268 men and 87,349 women) demonstrated comparable levels of protection against major vascular events and stroke in men and women [34]. There was no difference in effectiveness of different drug classes in men and women for cardiovascular events, although women may derive greater benefit from calcium antagonists than ACE-inhibitors for stroke prevention. The relevance of age and race for stroke protection in women was highlighted by a Cochrane review of 23,000 women [35]. In women aged 55 years and above (of whom 90% were white), antihypertensive therapy yielded a 38% risk reduction in both fatal and non-fatal cerebrovascular events (27–47%). In comparison, a 41% (8–63%) reduction was seen in younger women, and a 53% (29–69%) reduction was observed in African American women.

### Carotid endarterectomy

There is evidence of a different risk-benefit ratio in men and women for carotid endarterectomy (CEA). Studies have demonstrated that women are less likely to undergo CEA than men, likely due to lower incidence of high-grade symptomatic carotid stenosis [36, 37]. In addition, the number needed to treat to prevent 1 stroke in 5 years following CEA was 9 for men versus 36 for women [38]. A pooled analysis reported comparable benefits in stroke risk reduction from CEA in patients with ≥70% carotid stenosis, with a 5-year absolute risk reduction (ARR) in ischaemic stroke of 15.1% (*p* = 0.007) in women and 17.3% (*p* < 0.001) in men. Although there was a greater perioperative risk of death in women (2.3% versus 0.8% in men, *p* = 0.002). In this analysis, in the setting of 50–69% stenosis, CEA only provided benefit for men (men: ARR 10.0%, *p* = 0.02; women: ARR 3.0%, *p* = 0.94) [39]. It is important to note however, that women made up under 1/3 of participants in these trials. Pathological evaluation of CEA specimens established that plaques from women have a less inflammatory and more stable phenotype and are perhaps less likely to lead to a recurrent event [40].

### Contraceptive and hormone replacement therapy

It is common for stroke to occur in women taking either hormonal based contraceptive therapy or hormone replacement therapy (HRT). There is no evidence from randomised trials that HRT reduces the risk of ischaemic or haemorrhagic stroke. This was reviewed in the recent European Stroke Organisation guidelines on stroke in women [41]. On meta-analysis of data from 6 trials and approximately 45,000 participants, there was no difference in rate of ischaemic stroke (OR 0.97, 95% CI 0.66 to 1.41). On meta-analysis of data from 5 trials and approximately 35,000 participants, there was no difference in the rate of haemorrhagic stroke (OR 0.75, 95% CI 0.49 to 1.15). The use of oestrogen containing combined oral contraceptive treatment has been associated with an increased risk of both myocardial infarction and stroke. In a meta-analysis of 14 studies, the risk of stroke was increased more than two-fold [42]. The risk of stroke with oral contraceptives is higher in people with other cardiovascular risk factors including smoking, increasing age, and in particular with migraine with aura [43]. For example, in this study, the risk of stroke was increased 7-fold in women with migraine with aura taking the contraceptive pill, although there was considerable uncertainty in this estimate. The increased risk is dose related [44] with a reported increase in odds of stroke of 1.2 every 10-μg increment in oestrogen dose. There is less certainty regarding progestogen only contraceptives and they may not increase risk, but data are limited [45]. However, they are the more appropriate choice should oral contraception be required in a high-risk person.

### Treatment of ischaemic stroke in pregnancy and the post-partum period

Stroke during pregnancy is fortunately rare but occurs in approximately 34 of every 100,000 deliveries [46]. It may also be increasing due to a rise in average maternal age at birth. Physiological changes in pregnancy such as a hypercoagulable state, haemodynamic changes and development of hypertensive disorders contribute to this risk [47]. Management of stroke in pregnancy is complicated by a lack of evidence. Pregnancy is a typical exclusion from clinical trials, there is limited experience of use of medication in pregnancy, and treatment for ischaemic stroke is based around reperfusion, which typically requires thrombolytic or antithrombotic drug therapy.

The effect of intravenous thrombolytic therapy in pregnant women was recently reviewed in the European Stroke Organisation guidelines on stroke in women [41]. Evidence was limited to a single observational registry study [46] and case reports of 33 participants. The summary of case reports showed that there was an ‘improved or good recovery’ in 32 of 33 women treated, a healthy baby in 28 of 32 cases, abortion or termination in 4 cases, 3 cases of intracerebral haemorrhage and 1 case of intrauterine bleeding. The level of certainty was very low and publication bias was suspected. The registry study used data from the US Get With The Guidelines (GWTG) Stroke Registry [46]. Outcomes were generally comparable in pregnant and non-pregnant women, although symptomatic ICH rates tended to be higher. This led to a guideline consensus statement that “pregnant women with acute disabling ischaemic stroke, who otherwise meet eligibility criteria, can be treated with intravenous thrombolysis after appropriately assessing the benefit/risk profile on an individual basis.” The evidence base for mechanical reperfusion in pregnancy was also limited to case reports, with similar uncertainty regarding its effect. Independent outcomes were achieved in all treated participants from 23 case reports and a healthy baby was born in 18 cases. This led to a guideline consensus statement that pregnant women with acute ischaemic stroke and large vessel occlusion, who otherwise meet eligibility criteria, can be treated with mechanical thrombectomy after appropriate assessment of the benefit/risk profile on an individual basis. In addition, it was stated that mechanical thrombectomy should be preferred over intravenous thrombolysis.

There is a similar paucity of data regarding use of reperfusion treatments in the post-partum period. During the development of the European Stroke Organisation guidelines on stroke in women [41], only 2 case reports were found involving intravenous thrombolysis (at least 10-days post-partum) and 5 were found for mechanical thrombectomy. This led to expert consensus statements that intravenous thrombolysis can be considered at 10 or more days post-partum and that mechanical thrombectomy could be considered in preference to intravenous thrombolysis if available.

## Conclusions

We found there are differences in incidence of stroke between men and women. These likely differ by age but stroke remains an important cause of death and disability in both men and women. There is concerning evidence that treatments such as anticoagulation and thrombolytic therapy are underused in women, but this gap does seem to be narrowing. There are some differences in relative effectiveness of some treatments between men and women but for most aspects of stroke care benefit is clear in both men and women. The emphasis must be on ensuring more equitable access and greater representation of women in clinical trials. There is limited evidence to inform decision making during pregnancy but guidelines on this topic are now available to support clinicians.
